# Supplementary material for: Induction of Extrinsic Apoptotic Pathway in Pancreatic Cancer Cells by Apteranthes europaea Root Extract
Source: Int J Mol Sci. 2025 Oct 21;26(20):10221. doi: 10.3390/ijms262010221 (PMC12565304; doi:10.3390/ijms262010221)
Supplement: Supplementary file 1 [file ijms-26-10221-s001.zip › ijms-3852934-supplementary.pdf]

Supplementary Materials

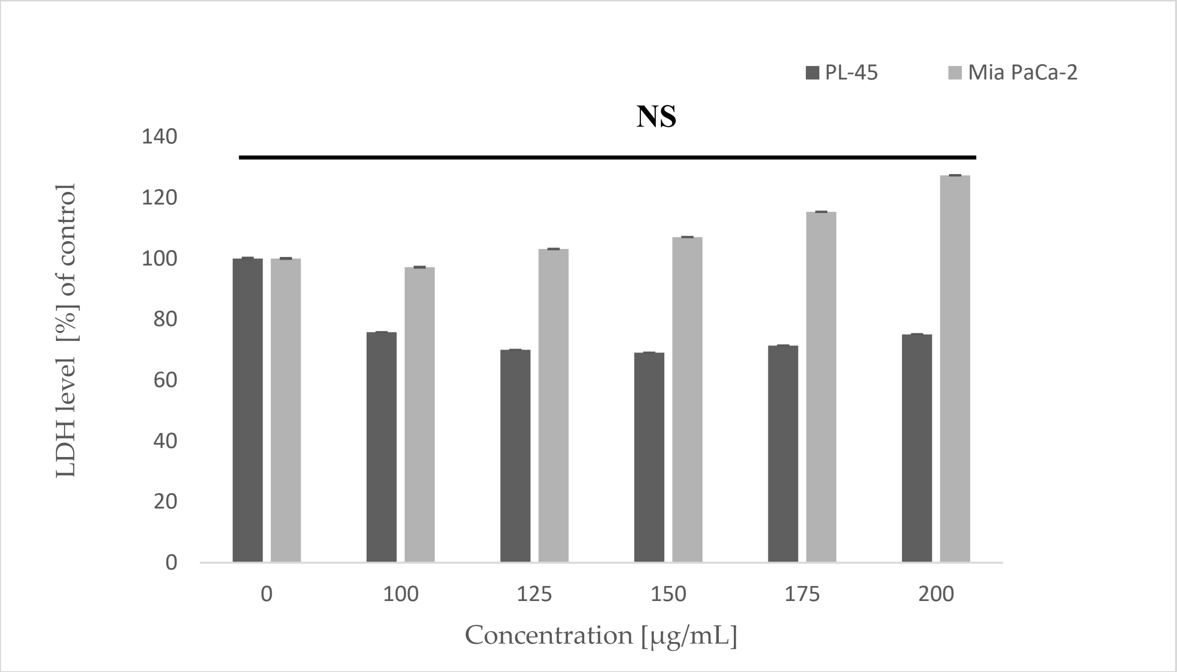

Figure S1. The effect of *A. europaea* extract on LDH levels on PL45 and Mia PaCa-2 cell lines. Cells were treated with different doses (0-200 µg/mL) of *A. europaea* extract for 24 h. At the end of treatment, samples of media from control and treated cells were tested for LDH release. The data presented are an average of three independent experiments each conducted in triplicates (mean ±SE) and are expressed as percentages of respective controls. Statistical significance was determined by a two-tailed student's t-test $P<0.05$ (treatment vs. control for each concentration); NS: Not significant for both cell lines.
